# Preparation of Stable Superhydrophobic Coatings on Wood Substrate Surfaces via Mussel-Inspired Polydopamine and Electroless Deposition Methods

**DOI:** 10.3390/polym9060218

**Published:** 2017-06-12

**Authors:** Kaili Wang, Youming Dong, Wei Zhang, Shifeng Zhang, Jianzhang Li

**Affiliations:** 1 Key Laboratory of Wood-Based Materials Science and Utilization, Beijing Forestry University, No. 35 Tsinghua East Road, Haidian District, Beijing 100083, China; wangkaili212@163.com (K.W.); dongyouming@bjfu.edu.cn (Y.D.); zhangweishe@126.com (W.Z.); 2 Beijing Key Laboratory of Wood Science and Engineering, Beijing Forestry University, No. 35 Tsinghua East Road, Haidian District, Beijing 100083, China

**Keywords:** electroless deposition, mussel-inspired polydopamine, wood surface, superhydrophobicity, stability

## Abstract

Mussel-inspired polydopamine (PDA) chemistry and electroless deposition approaches were used to prepare stable superhydrophobic coatings on wood surfaces. The as-formed PDA coating on a wood surface exhibited a hierarchical micro/nano roughness structure, and functioned as an “adhesive layer” between the substrate and a metallic film by the metal chelating ability of the catechol moieties on PDA, allowing for the formation of a well-developed micro/nanostructure hierarchical roughness. Additionally, the coating acted as a stable bridge between the substrate and hydrophobic groups. The morphology and chemical components of the prepared superhydrophobic wood surfaces were characterized by scanning electron microscopy (SEM), Fourier transform infrared (FT-IR) spectroscopy, and X-ray photoelectron spectroscopy (XPS). The PDA and octadecylamine (OA) modified surface showed excellent superhydrophobicity with a water contact angle (CA) of about 153° and a rolling angle (RA) of about 9°. The CA further increased to about 157° and RA reduced to about 5° with the Cu metallization. The superhydrophobic material exhibited outstanding stability in harsh conditions including ultraviolet aging, ultrasonic washing, strong acid-base and organic solvent immersion, and high-temperature water boiling. The results suggested that the PDA/OA layers were good enough to confer robust, degradation-resistant superhydrophobicity on wood substrates. The Cu metallization was likely unnecessary to provide significant improvements in superhydrophobic property. However, due to the amazing adhesive capacity of PDA, the electroless deposition technique may allow for a wide range of potential applications in biomimetic materials.

## 1. Introduction

Superhydrophobic surfaces have recently attracted significant attention in both scientific and industrial sectors for potential applications in nonwetting, self-cleaning, anti-fogging, anti-icing, anti-corrosion, oil-water separation, and drag-reduction [1,2,3,4,5,6,7]. Artificial superhydrophobic surfaces have been broadly designed and constructed by learning from examples in nature, such as lotus leaves with superhydrophobicity. According to the classical theories (i.e., the Wenzel and Cassie-Baxter models) [8], the combination of the surface chemical composition and the topographic structure are responsible for the superhydrophobic property.

Wood, as an environmentally friendly and aesthetically pleasing biopolymer material, is widely used in the daily lives of humans for various applications, such as construction, furniture, and indoor decoration. However, wood is susceptible to the absorbance of water and moisture, which renders wood vulnerable to fungi and dimensional instability, strongly reducing the durability and service lifetime [9]. The addition of superhydrophobic surfaces on a wood substrate has great potential to address these problems and extend the service life of the resulting products [10].

Various methods have been developed to design and construct superhydrophobic wood surfaces by creating lotus-leaf-like hierarchical structures, including the sol-gel technique, hydrothermal method, solution-immersion, chemical vapor deposition, layer-by-layer assembly, and plasma treatment [11,12,13,14,15,16]. However, these traditional methods have some limitations. For example, some approaches are tedious, require specialized experimental equipment, or harsh processing conditions. Furthermore, they limit the types, sizes, and shapes of substrates, or even degraded the wood’s original components and structures. They may result in materials with poor environment durability, limiting their practical applications [17].

A typical reduced copper nanoparticle approach was conducted under a weak alkaline and ambient-temperature condition [18,19]. This method can be used for applying material to a wood surface to construct a hierarchical roughness structure with slight degradation of the wood components and structures, thus retaining the pristine strength of the wood. However, the formed Cu nanoparticles likely bind with the hydroxyl groups of the wood cellulose through weak hydrogen bonding, which may be lost from the wood substrate surfaces during the long-term service process, resulting in unstable superhydrophobicity.

Inspired by the amazing adhesive ability of marine mussels, a novel method to metallize nonconductive materials was proposed by Lee and co-workers. This method uses an intermediate, polydopamine (PDA) coating to act as an “adhesive layer” between the substrate and a metallic film by the metal chelating ability of the catechol moieties on PDA [20]. The combination of a PDA-based functionalization together with electroless coating ensures a simple, versatile, scalable, and low-cost metal coating strategy [21]. Compared to the electrochemical deposition or chemical/physical vapor deposition processes, this method is easy to perform and does not depend on expensive instruments. Most importantly, this approach is suitable for different kinds of substrates, irrespective of their shapes or conductivities [22]. In addition, the free catechol groups on the PDA layer have diverse secondary reactions, typically reacting with amino-containing molecules through Michael addition or Schiff base reactions [23]. Accordingly, this method allows the design of hierarchical structures to mimic the lotus-leaf-like surfaces with stable superhydrophobic properties.

In this paper, we propose a novel method to prepare superhydrophobic wood surfaces with excellent environment durability. To the best of our knowledge, there are no previous reports on the preparation of superhydrophobic wood surfaces through mussel-inspired dopamine chemistry and electroless deposition methods, which use the PDA coating as an “adhesive layer” between the wood surface and the metallic film. In this procedure, dopamine spontaneously self-polymerized into PDA under basic conditions, and tightly adhered onto the wood surface. Then, copper was electrolessly deposited onto PDA-modified wood surfaces via chelation between the catechol moieties on PDA and the as-reduced copper species, resulting in the creation of micro/nano hierarchical structures on wood surfaces. Finally, grafting of amine-containing hydrophobic groups onto as-formed rough surfaces occurred through a Michael addition or a Schiff base reaction, and the lotus-leaf-like superhydrophobic surfaces were successfully prepared. The electroless deposition method presents the following points of innovation and significant advantages. (1) This is the first demonstration of the preparation of superhydrophobic wood surfaces by a mussel-inspired dopamine chemistry and an electroless deposition of a copper species method via PDA as an intermediate layer. (2) The whole procedure was conducted under mild conditions without complex instruments, and did not destroy the intrinsic structure of the wood. (3) The as-prepared superhydrophobic surfaces showed excellent environmental durability in various harsh conditions, including strong acid/base, organic solvent, boiling water, ultrasonic washing, and UV radiation. (4) This surface modification method can be applied to a wide range of material surfaces, irrespective of their scale and shapes, due to the strong adhesive ability of the PDA layer.

## 2. Materials and Methods

### 2.1. Materials

Defect-free and straight-grained sapwood portions of fast-growing poplar wood (Populus tomentosa Carr.) were manufactured into blocks with a dimension of 3 × 20 × 20 mm^3^ (radial × tangential × longitudinal). Dopamine hydrochloride (99% purity), tris (hydroxymethyl) aminomethane (Tris, 99% purity), and octadecylamine (99% purity) were purchased from Tianjin Heowns Biochem Co., Ltd (Tianjin, China). Cupric chloride dehydrate (CuCl_2_·2H_2_O, 99.99% purity) and borane dimethylamine complex (DMAB, 96% purity) were purchased from Shanghai Macklin Biochemical Co., Ltd (Shanghai, China). Ethylenediamine tetraacetic acid (EDTA, 99.5% purity), boric acid (H_3_BO_3_, 99.5% purity), sodium hydroxide, anhydrous ethanol, toluene, acetone, hydrochloric acid, sodium hydroxide, *n*-hexane, and *N*,*N*-Dimethylformamide (DMF) were purchased from Beijing Chemical Works (Beijing, China).

### 2.2. Preparation of Superhydrophobic Wood Surfaces

All wood blocks were Soxhlet-extracted with a mixture of toluene/ethanol/acetone (4:1:1 *v*/*v*/*v*) for 12 h, and dried in an oven at 103 ± 2 °C until a constant weight was reached. The immersion solution (2.0 mg/mL) was prepared by dissolving dopamine in Tris-HCl (10 mM) buffer solution with a pH value of 8.5. The wood blocks were immersed into the solution and stirred for 24 h at 60 °C, allowing for the deposit of a PDA layer on the surface of the wood blocks. The as-obtained wood samples were washed with deionized water several times and then dried in an oven at 60 °C. The PDA-coated wood samples were metallized by immersion into an electroless copper bath for 12 h. The bath consisted of 50 mM CuCl_2_, 50 mM EDTA, and 100 mM H_3_BO_3_, buffered to pH 7.0 with NaOH, with the addition of 100 mM DMAB to initiate electroless deposition. The wood samples were washed with distilled water several times and dried at 60 °C. The PDA-coated and PDA/Cu coated wood samples were soaked in octadecylamine ethanol solution (1:100 *v*/*v*) for the reaction at 30 °C for 24 h, and then washed with ethanol several times and dried at 60 °C. The superhydrophobic wood surfaces with a lotus-leaf-like hierarchical structure were prepared. The formed superhydrophobic wood samples were labelled as PDA/Wood or PDA/Cu/Wood. The Octadecylamine modified wood, labelled as OA/Wood, was used as a control sample.

### 2.3. Characterizations

Scanning electron microscopy (SEM) images were acquired using the Quanta FEG 650 instrument (FEI, Hillsboro, OR, USA) operated at an accelerating voltage of 15 kV.

The X-ray photoelectron spectroscopy (XPS) with a K-Alpha X-ray photoelectron spectrometer (Thermo Fisher Scientific Co., Ltd, Shanghai, China) was operated at room temperature with monochromatic Al Kα radiation (1486.6 eV).

The superhydrophobic wood surfaces were milled to a 200-mesh particle size and embedded into potassium bromide (KBr) pellets at a weight ratio of 1:70. The pellets were then analyzed with an FTIR device (Nicolet 6700 Thermo Scientific, Madison, WI, USA) in a range of 4000 to 400 cm^−1^ at 4 cm^−1^ resolution for 32 scans.

Contact angles (CAs) were measured with a 3 µL deionized water droplet on a Dataphysics OCA 20 (Dataphysics, Filderstadt, Germany) instrument at room temperature. All the CAs were reported by averaging the values obtained at six different points on the longitudinal surfaces of wood samples, and the ellipse fitting modes were used to fit the shapes of water droplets.

To examine macroscopically rough superhydrophobic wood surfaces, the rolling angles (RAs) were measured following the procedure reported in the reference [24]. A water droplet of defined volume (about 10 μL) was released onto the wood surface, and the critical angle of inclination at which the wood samples needed to be tilted until the droplet rolled off the surface was recorded as the rolling angle.

The surface free energy was calculated by the CAs of two test liquids, namely, distilled water, and diiodomethane. The specifications of their surface tension and components are shown in Table 1.

The geometric mean equation (OWRK method) based on Young’s equation $\gamma_{s}=\gamma_{L}cos\theta +\gamma_{SL}$ was used to evaluate the surface free energy, where $\gamma_{s}$ is the surface tension of a solid, $\gamma_{L}$ is the surface tension of the liquid, $\gamma_{SL}$ is the surface tension of the solid-liquid interface, and θ is the CA between the solid (S) and liquid (L).

The OWRK method [25] uses the following equation:

(1) $$\gamma_{L}\left(1+cos\theta \right)=2\sqrt{\gamma_{S}^{d}\gamma_{L}^{d}}+2\sqrt{\gamma_{S}^{p}\gamma_{L}^{p}}$$

where $\gamma_{L}$ is the surface tension of the liquid, and θ is the CA between the solid (S) and the liquid (L). $\gamma_{S}^{d}$ and $\gamma_{S}^{p}$ are the dispersion and polar components in the surface free energy of the solid (mJ/m^2^), respectively, and $\gamma_{L}^{d}$ and $\gamma_{L}^{p}$ are the dispersion and polar components in the surface free energy of the liquid (mJ/m^2^), respectively. 

Environmental durability test:

Ultrasonic washing test: the superhydrophobic wood samples were submerged in distilled water six times for a total of 1 h under ultrasonication (40 kHz frequency, 100 W). The samples were collected at certain intervals and dried in an oven at 100 °C for 3 h followed by the CA measurement. Water boiling test: the wood samples were boiled with water for 2 h, oven-dried, and then the CAs were measured. Chemical durability test: the wood samples were immersed into an HCl solution (pH = 2), NaOH solution (pH = 12), and various organic solvents for 24 h, and the CAs were measured. UV radiation test: the superhydrophobic wood samples were placed in an ultraviolet aging test chamber (Beijing Beifang Lihui Instrument Equipment. Co., Ltd, Beijing, China) for a week (power: 40 W; radiation wavelength: 340 nm).

## 3. Results and Discussion

### 3.1. Preparation Process and Reaction Mechanism

**Scheme 1:**
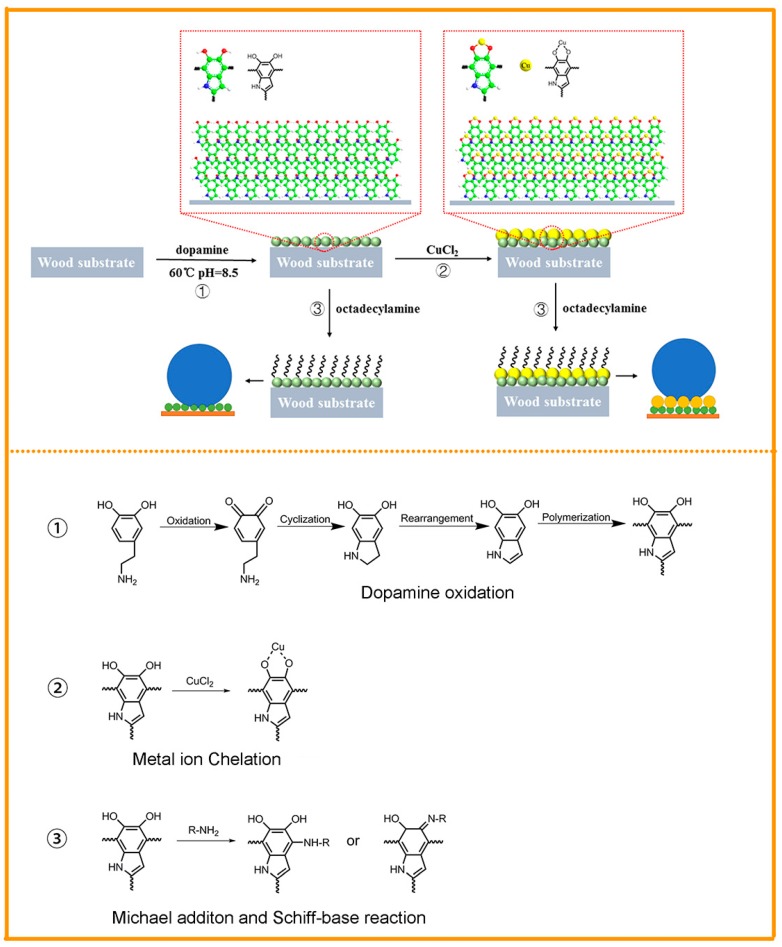
Preparation for the superhydrophobic surface and reaction mechanism. ① Dopamine oxidation polymerization process; ② Electroless metallization process: the catechol groups chelating copper species; ③ Grafting NH-terminated long-chain alkyls (octadecylamine) onto as-formed PDA coating through a Michael-addition and Schiff-base reactions.

The typical procedure of preparing the superhydrophobic surface is shown in Scheme 1. In an alkalescent environment, dopamine spontaneously polymerized into PDA, and strongly adhered on the wood substrate surface. As shown in the Wang and co-workers study [26], the surfaces of PDA coatings became rougher since the PDA particles grew faster and formed bigger nodules when increasing the reaction temperature to accelerate the reaction. Based on this, the dopamine self-polymerization reaction was conducted at 60 °C, which formed a micro/nano hierarchical roughness structure on wood surface, and further modified by grafting long-chain alkyls, resulting in a stable superhydrophobicity. In the aqueous solution of CuCl_2_, EDTA, H_3_BO_3_, and DMAB, Cu^2+^ was reduced to Cu^0^ through the reaction:

2(CH_3_)_2_NHBH_3_ + 6H_2_O + 3Cu^2+^ → 2(CH_3_)_2_NH + 2H_3_BO_3_ + 6H^+^ + 3Cu + 3H_2_

The catechol groups in the PDA coating exhibited a strong chelating capacity towards copper species, which promoted metal deposition on the wood substrate surfaces during the electroless metallization. The PDA/Cu hybrid coating endowed wood surfaces with a well-developed micro/nano hierarchical roughness. Then, grafting long-chain alkyls onto as-formed hierarchical surfaces through a Michael-addition and Schiff-base reactions achieved lotus-leaf-like surfaces with superhydrophobicity. 

Chang et al. prepared superhydrophobic coatings on wood surfaces using silica-polymer nanocomposites, and the resultant CAs showed a decreasing tendency from about 148° to 135° with increased leaching cycles [10]. Liu et al. constructed superhydrophobic wood surfaces via a hydrothermal process; however, this approach damaged the wood substrate structure and components due to the harsh environment [12]. In the present study, the whole process of electroless deposition-based PDA chelation was conducted under mild conditions, avoiding damaging the intrinsic structure of the wood substrate. The as-prepared superhydrophobic wood surfaces showed excellent stability in harsh conditions due to the strong chelating force formed between the PDA layers and the Cu films. 

### 3.2. Micromorphology and Chemical Component Analysis

**Figure 1:**
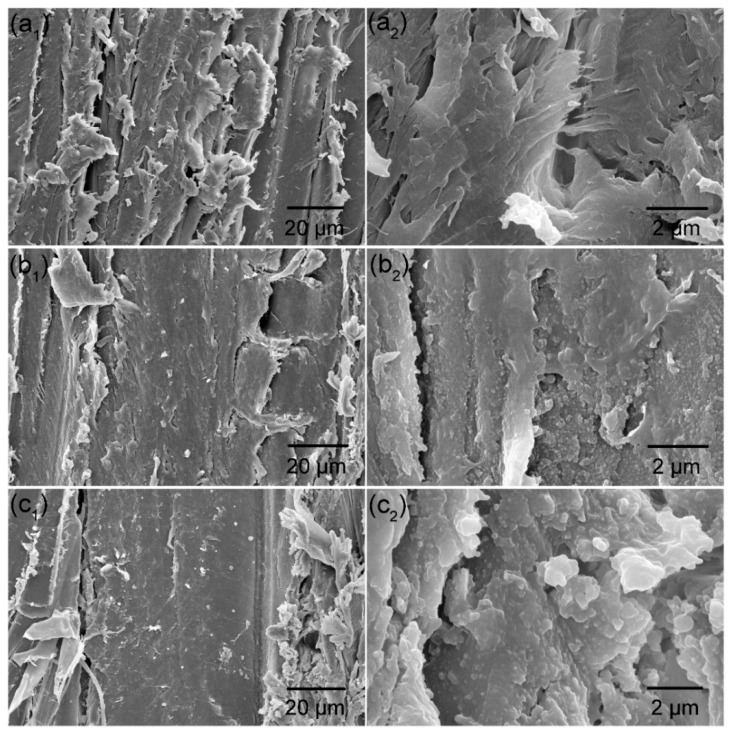
SEM observations of tangential sections of (**a**) control wood; (**b**) PDA/Wood; and (**c**) PDA/Cu/Wood at different magnifications.

Figure 1 shows the surface morphologies of the control wood, PDA/Wood, and PDA/Cu/Wood samples at different magnifications. The avulsed lamellar structures of the wood cell walls were apparent in the control wood, forming a roughness structure at the microscale level (Figure 1a). After coating with dopamine in a mildly alkaline environment at 60 °C for 24 h, a much rougher PDA layer composed of aggregated PDA particles was observed on the wood surface (Figure 1b). The excellent adhesion ability and reactivity of PDA allowed the electroless deposition of copper species by dipping the PDA-coated wood samples into the electroless bath. After the Cu metallization, a slight change but still rough micro/nano hierarchical structures were observed on the PDA-coated wood surfaces (Figure 1c). The surface morphology models of the two kinds of superhydrophobic samples are illustrated in Scheme 1. The PDA assemblies and deposited Cu nanoparticles and aggregates served as building blocks to create micro/nano multiscale hierarchical structures on wood substrate surfaces. After grafting long-chain alkyl groups, the mimetic lotus leaf surface with superhydrophobicity was successfully prepared.

**Figure 2:**
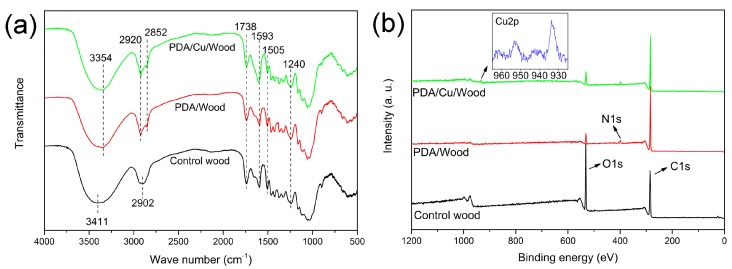
FTIR spectrum (**a**), and XPS spectra (**b**) for the control wood, PDA/Wood, and PDA/Cu/Wood samples, the inset picture is the Cu2p core-level spectra for PDA/Cu/Wood.

The FTIR spectra of the control wood, PDA/Wood, and PDA/Cu/Wood samples are shown in Figure 2a. For the control wood, the prominent band at 3411 cm^−1^ was assigned to the stretching vibration of the OH groups, and the band at 2902 cm^−1^ was assigned to C–H stretching vibrations. The bands at 1738, 1593, 1505, and 1240 cm^−1^ were assigned to the C=O stretching of the acetyl groups, the aromatic skeletal vibration of lignin, and C–O stretching of the guaiacyl ring, respectively [27]. For the PDA-coated wood samples, the characteristic peak at 3411 cm^−1^ for the OH groups shifted to 3354 cm^−1^, likely because of the hydrogen bond formation between PDA and wood hydroxyl groups [28,29]. Two prominent peaks at 2920 cm^−1^ and 2852 cm^−1^ for the PDA/Wood and PDA/Cu/Wood were assigned to –CH_3_ and –CH_2_ asymmetrical stretching vibrations and symmetrical stretching vibrations from long alkyl chains, respectively [30]. According to previous studies [31,32], the PDA prominent peaks at 1510, 1600, and 1274 cm^−1^ were assigned to the N–H scissoring vibrations, stretching from the indole ring, and C–O stretching from phenolic moieties, respectively. However, the FTIR spectroscopy did not provide clear evidence of the presence of PDA, and even less evidence of Cu on the coated samples was observed. This was probably because wood is a complex polymer containing, among others, polyphenolic moieties, which likely shared many signals with (poly)dopamine. Furthermore, PDA layers deposited from tris buffers and aerobic oxidation were usually just a few nanometers thick, so, they were likely negligible as a weight fraction of the sample analyzed by FTIR. The surface elemental analysis for the wood samples was conducted by XPS, as shown in Figure 2b. The control wood only showed C and O signals, but the N signal appeared after PDA coating of wood surfaces. As expected, the characteristic Cu peak appeared in the spectrum of the PDA/Cu/Wood, (the inserted picture for a blow-up of the Cu2p peak region) and the at. % was 1.08%, indicating that Cu particles were successfully deposited on the PDA coated wood surfaces. These results indicate that the PDA was successfully coated to the wood substrate surfaces, and the Cu was subsequently immobilized onto the PDA layer by the electroless deposition process, and the long-chain alkyl groups were grafted onto the PDA coating.

### 3.3. Superhydrophobic Property and Stability

**Figure 3:**
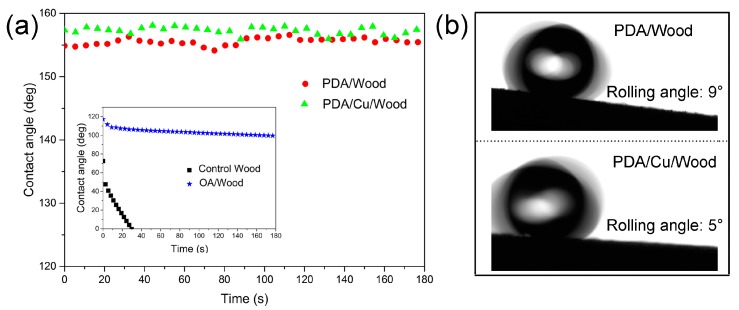
Superhydrophobic performance characterization, (**a**) contact angles as a function of time for control wood and OA/Wood (inset), PDA/Wood, and PDA/Cu/Wood; (**b**) the profile of water droplets rolled down on the inclined surface of the PDA/Wood and PDA/Cu/Wood samples.

Figure 3a shows the changes in CAs on the surface of the wood samples over time. The CAs of the control wood decreased rapidly in 30 s, and the CAs of OA/Wood decreased from about 117° to 100° in 180 s, which shows hydrophobic property. In contrast, the CAs on the PDA/Wood and PDA/Cu/Wood surfaces showed no obvious change for this time period, and all remained over 150° after 180 s. The mean CAs values of PDA/Cu/Wood samples (157°) were slightly larger than those of PDA/Wood (153°). Figure 3b displays the change of RAs for PDA/Wood and PDA/Cu/Wood, which decreased from 9° to 5°. The slight change of CAs and RAs of PDA/Wood and PDA/Cu/Wood were likely assigned to the different surface micromorphologies. However, the CAs significance tests between PDA/Wood and PDA/Cu/Wood samples were conducted, and its *p*-value was 0.09, which showed that the change was not statistically significant. Therefore, it was enough for PDA/OA layers to endow with superhydrophobic performance on wood surfaces, and the Cu metallization only slightly changed the micro/nano hierarchical roughness structures. However, it is likely unnecessary to provide significant improvements in superhydrophobicity.

**Figure 4:**
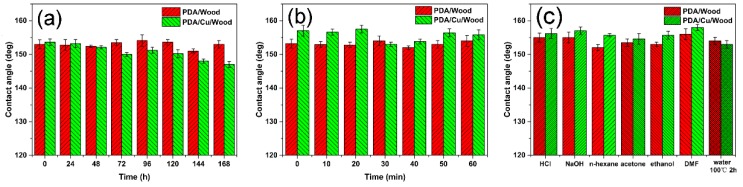
Environment durability characterization of PDA/Wood and PDA/Cu/Wood. (**a**) CAs as a function of UV radiation exposure time; (**b**) CAs as a function of time of ultrasonic washing in water; (**c**) CAs for wood samples soaked into different chemical solutions for 24 h.

Figure 4a shows the superhydrophobic stability under long exposure to UV light. The CAs of the PDA/Wood and PDA/Cu/Wood surfaces remained about 150° after 168 h of UV radiation, indicating that the samples had outstanding ability to withstand UV radiation. The CAs changes of superhydrophobic wood surfaces after ultrasonic washing (40 kHz, 100 W) within 60 min were evaluated (Figure 4b). All were maintained above 150° after the ultrasonic washing, demonstrating the excellent adhesive property of the PDA layer. The observed CA changes were characterized after the immersion of superhydrophobic wood samples into various chemical reagents (separate solutions of HCl, pH = 2, NaOH, pH = 12, *n*-hexane, acetone, ethanol, and DMF) for 24 h, and 100 °C boiling water for 2 h. The CAs all remained above 150°, indicating great chemical resistance. All these results demonstrated that the as-prepared bionic superhydrophobic wood surfaces all exhibited excellent stability under harsh environments.

Wang et al. fabricated superhydrophobic wood surfaces by drop-coating a mixed solution comprising of modified silica particles and polystyrene emulsion. The CAs of wood sample showed a slight decrease from 153° to 148° after undergoing 25 min ultrasonic washing [33]. Liu et al. used a convenient solution-immersion method to prepare superhydrophobic wood surfaces from potassium methyl siliconate, and found that the CAs decreased dramatically after the wood was soaked in a strong alkali solution [13]. Cai et al. fabricated a superhydrophobic wood surface through a solution-immersion process with γ-aminopropyltriethoxysilane and lauric acid. The CAs decreased to 0° after treatment with acetone, chloroform, and DMF [34]. Although these methods can achieve superhydrophobicity on wood surfaces, the produced materials showed poor environmental durability. In the present study, the mussel-inspired dopamine chemistry and electroless deposition process can both form stable and durable superhydrophobic coatings, with outstanding performance for resisting various harsh environments.

**Figure 5:**
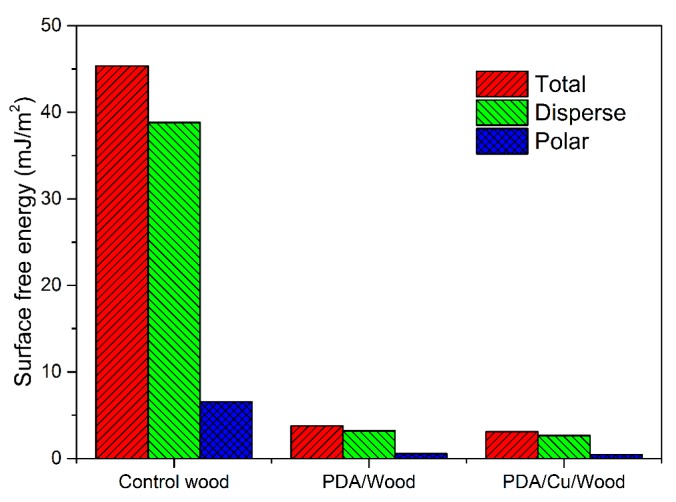
Surface free energy (including total surface free energy, dispersion components and polar components) of control wood, PDA/Wood, and PDA/Cu/Wood samples obtained by the OWRK method.

Since the standard deviations of the determined CAs were small and the surface free energy reduction from the control wood to the modified wood was significant, the initial CAs were used to replace the equilibrium CAs for the calculation of the surface free energy [30]. The surface free energy results with the OWRK method are provided in Figure 5. The surface free energy of the control wood was 45.35 mJ/m^2^, which was in accordance with the reported values ranging from 40 to 60 mJ/m^2^ [35]. The surface free energy (including the polar and dispersion components) remarkably decreased from 45.35 to 3.76 mJ/m^2^ for PDA/Wood, and to 3.10 mJ/m^2^ for PDA/Cu/Wood, indicating that the PDA/Cu/Wood sample surface had lower free energy, due to the as-constructed micromorphology differences between PDA/Wood and PDA/Cu/Wood. However, the significance tests results (*p*-values was 0.08) of surface free energy between PDA/Wood and PDA/Cu/Wood samples indicated that the electroless deposition process did not significantly decrease the surface free energy. The superhydrophobicity can be explained by the as-constructed hierarchical roughness surfaces as well as the reduced free energy.

To further understand the effect of as-prepared micro/nano hierarchical structure for PDA/Wood and PDA/Cu/Wood samples on their superhydrophobic performance, the Cassie-Baxter equation was employed, which is generally applicable to a hierarchical or heterogeneous substrate [36].

(2) $$\cos\theta_{a}=f\left(\cos\theta +1\right)-1$$

where θ and $\theta_{a}$ represent the CAs on smooth and rough surfaces, respectively, *f* is the apparent area fraction of the solid surface in contact with liquid, and 1 − *f* is the fraction of trapped air in contact with liquid at the surface. In this equation, θ is a constant value for a certain material, and the CA of water on a long-chain alkyl modified smooth surface was 94.8° [37]. By calculation, the trapped air fraction in contact with water for PDA/Wood was 0.88 and for PDA/Cu/Wood was 0.91. A higher air fraction contributed to larger CAs and smaller RAs. Nevertheless, the statistical tests of trapped air fraction between PDA/Wood and PDA/Cu/Wood was carried out, and the *p*-value was 0.09, indicating that there was not a statistical significance between them. Microstructure models were proposed as shown in Scheme 1 based on the slight difference of the surface micromorphologies for PDA/Wood and PDA/Cu/Wood samples, as shown in Figure 1. After wood samples were immersed into the dopamine, a thin and rough PDA layer was coated onto wood surfaces with aggregated PDA particles, which formed a hierarchical roughness. With the deposition of Cu particles on the PDA-coated wood surfaces, the micro/nano hierarchical structures were further well-developed. These two kinds of roughness surfaces were both suitable for a stable superhydrophobic property on wood surface after grafting hydrophobic groups. However, the PDA/OA layers on wood surfaces were good enough to confer robust, degradation-resistance superhydrophobicity, while the Cu metallization was likely unnecessary to provide any significant improvements in this respect. In terms of the electroless deposition approach based on the outstanding adhesion ability and reactivity of the mussel-inspired PDA coating, it is efficient, simple, and mild, allowing for extensive applications.

## 4. Conclusions

In this study, the novel and simple mussel-inspired dopamine chemistry and electroless deposition approach was developed to prepare superhydrophobic surfaces. The as-formed PDA coatings acted as an intermediate layer that joined the substrate and metallic film, synergistically formed well-developed micro/nanostructure hierarchical roughness and also bridged the hydrophobic groups on the as-formed surfaces. The superhydrophobic surfaces showed excellent stability against various harsh environments including ultraviolet aging, ultrasonic washing, strong acid/base, organic solvent, and high-temperature water boiling. It is worth mentioning that the PDA/OA layers are good enough to confer robust, degradation-resistant superhydrophobicity to wood substrates. The Cu metallization is likely unnecessary to provide any significant improvement in superhydrophobic performance. However, the electroless deposition approach based on the outstanding adhesion ability and reactivity of the mussel-inspired PDA coating is efficient, simple, mild, does not require specialized instruments, and can be used for many different materials, irrespective of the styles, shapes, and sizes of substrates, allowing for extensive applications.

## Figures and Tables

**Table 1:** Surface Tension and Components of the Test Liquids.

Type of Liquid Reference	Surface Free Energy (mJ/m^2^)				
	$\gamma_{L}$	$\gamma_{L}^{LW}$ ($\gamma_{L}^{d}$)	$\gamma_{L}^{AB}$ ($\gamma_{L}^{P}$)	$\gamma_{L}^{+}$	$\gamma_{L}^{-}$
Distilled Water	72.8	21.8	51.0	25.5	25.5
Diiodomethane	50.8	50.8	0	0	0

## References

[1] Zimmermann J., Reifler F.A., Fortunato G., Gerhardt L.C., Seeger S. (2008). A simple, one-step approach to durable and robust superhydrophobic textiles. Adv. Funct. Mater..

[2] Fürstner R., Barthlott W., Neinhuis C., Walzel P. (2005). Wetting and self-cleaning properties of artificial superhydrophobic surfaces. Langmuir.

[3] Gao X., Yan X., Yao X., Xu L., Zhang K., Zhang J., Yang B., Jiang L. (2007). The dry-style antifogging properties of mosquito compound eyes and artificial analogues prepared by soft lithography. Adv. Mater..

[4] Kim P., Wong T.S., Alvarenga J., Kreder M.J., Adorno-Martinez W.E., Aizenberg J. (2012). Liquid-infused nanostructured surfaces with extreme anti-ice and anti-frost performance. ACS Nano.

[5] Liu H., Szunerits S., Xu W., Boukherroub R. (2009). Preparation of superhydrophobic coatings on zinc as effective corrosion barriers. ACS Appl. Mater. Interfaces.

[6] Guix M., Orozco J., García M., Gao W., Sattayasamitsathit S., Merkoçi A., Escarpa A., Wang J. (2012). Superhydrophobic alkanethiol-coated microsubmarines for effective removal of oil. ACS Nano.

[7] Xue Y., Chu S., Lv P., Duan H. (2012). Importance of hierarchical structures in wetting stability on submersed superhydrophobic surfaces. Langmuir.

[8] Cassie A., Baxter S. (1944). Wettability of porous surfaces. Trans. Faraday Soc..

[9] Mohammed-Ziegler I., Tánczos I., Hórvölgyi Z., Agoston B. (2008). Water-repellent acylated and silylated wood samples and their surface analytical characterization. Colloids Surf. A.

[10] Chang H., Tu K., Wang X., Liu J. (2015). Facile preparation of stable superhydrophobic coatings on wood surfaces using silica-polymer nanocomposites. BioResources.

[11] Wang S., Liu C., Liu G., Zhang M., Li J., Wang C. (2011). Fabrication of superhydrophobic wood surface by a sol-gel process. Appl. Surf. Sci..

[12] Liu M., Qing Y., Wu Y., Liang J., Luo S. (2015). Facile fabrication of superhydrophobic surfaces on wood substrates via a one-step hydrothermal process. Appl. Surf. Sci..

[13] Liu C., Wang S., Shi J., Wang C. (2011). Fabrication of superhydrophobic wood surfaces via a solution-immersion process. Appl. Surf. Sci..

[14] Ma M., Mao Y., Gupta M., Gleason K.K., Rutledge G.C. (2005). Superhydrophobic fabrics produced by electrospinning and chemical vapor deposition. Macromolecules.

[15] Renneckar S., Zhou Y. (2009). Nanoscale coatings on wood: Polyelectrolyte adsorption and layer-by-layer assembled film formation. ACS Appl. Mater. Interfaces.

[16] Xie L., Tang Z., Jiang L., Breedveld V., Hess D.W. (2015). Creation of superhydrophobic wood surfaces by plasma etching and thin-film deposition. Surf. Coat. Technol..

[17] Wu Y., Jia S., Qing Y., Luo S., Liu M. (2016). A versatile and efficient method to fabricate durable superhydrophobic surfaces on wood, lignocellulosic fiber, glass, and metal substrates. J. Mater. Chem. A.

[18] Frick C.P., Merkel D.R., Laursen C.M., Brinckmann S.A., Yakacki C.M. (2016). Copper-coated liquid-crystalline elastomer via bioinspired polydopamine adhesion and electroless deposition. Macromol. Rapid Commun..

[19] Plana D., Campbell A.I., Patole S.N., Shul G., Dryfe R.A. (2010). Kinetics of electroless deposition: The copper-dimethylamine borane system. Langmuir.

[20] Lee H., Dellatore S.M., Miller W.M., Messersmith P.B. (2007). Mussel-inspired surface chemistry for multifunctional coatings. Science.

[21] Mondin G., Wisser F.M., Leifert A., Mohamed-Noriega N., Grothe J., Dörfler S., Kaskel S. (2013). Metal deposition by electroless plating on polydopamine functionalized micro-and nanoparticles. J. Colloid Interface Sci..

[22] Zhao L., Chen D., Hu W. (2016). Patterning of metal films on arbitrary substrates by using polydopamine as a uv-sensitive catalytic layer for electroless deposition. Langmuir.

[23] Kang H., Song X., Wang Z., Zhang W., Zhang S., Li J. (2016). High-performance and fully renewable soy protein isolate-based film from microcrystalline cellulose via bio-inspired poly(dopamine) surface modification. ACS Sustain. Chem. Eng..

[24] Li S., Huang J., Chen Z., Chen G., Lai Y. (2017). A review on special wettability textiles: Theoretical models, fabrication technologies and multifunctional applications. J. Mater. Chem. A.

[25] Zhang S., Xia C., Dong Y., Yan Y., Li J., Shi S., Cai L. (2016). Soy protein isolate-based films reinforced by surface modified cellulose nanocrystal. Ind. Crops Prod..

[26] Wang Y., Shang B., Hu X., Peng B., Deng Z. (2017). Temperature control of mussel-inspired chemistry toward hierarchical superhydrophobic surfaces for oil/water separation. Adv. Mater. Interfaces.

[27] Dong Y., Yan Y., Zhang S., Li J., Wang J. (2015). Flammability and physical-mechanical properties assessment of wood treated with furfuryl alcohol and nano-SiO_2_. Eur. J. Wood Wood Prod..

[28] Salavagione H.J., Martínez G., Gómez M.A. (2009). Synthesis of poly(vinyl alcohol)/reduced graphite oxide nanocomposites with improved thermal and electrical properties. J. Mater. Chem..

[29] Xiong S., Wang Y., Yu J., Chen L., Zhu J., Hu Z. (2014). Polydopamine particles for next-generation multifunctional biocomposites. J. Mater. Chem. A.

[30] Wang K., Dong Y., Yan Y., Qi C., Zhang S., Li J. (2016). Preparation of mechanical abrasion and corrosion resistant bulk highly hydrophobic material based on 3-d wood template. RSC Adv..

[31] Kang S., Baginska M., White S.R., Sottos N.R. (2015). Core–shell polymeric microcapsules with superior thermal and solvent stability. ACS Appl. Mater. Interfaces.

[32] Luo R., Tang L., Wang J., Zhao Y., Tu Q., Weng Y., Shen R., Huang N. (2013). Improved immobilization of biomolecules to quinone-rich polydopamine for efficient surface functionalization. Colloids Surf. B.

[33] Wang C., Zhang M., Xu Y., Wang S., Liu F., Ma M., Zang D., Gao Z. (2014). One-step synthesis of unique silica particles for the fabrication of bionic and stably superhydrophobic coatings on wood surface. Adv. Powder Technol..

[34] Cai P., Bai N., Xu L., Tan C., Li Q. (2015). Fabrication of superhydrophobic wood surface with enhanced environmental adaptability through a solution-immersion process. Surf. Coat. Technol..

[35] Qin Z., Gao Q., Zhang S., Li J. (2014). Surface free energy and dynamic wettability of differently machined poplar woods. BioResources.

[36] Campos R., Guenthner A.J., Meuler A.J., Tuteja A., Cohen R.E., McKinley G.H., Haddad T.S., Mabry J.M. (2012). Superoleophobic surfaces through control of sprayed-on stochastic topography. Langmuir.

[37] Farzaneh M., Kulinich S., Volat C. (2004). Hydrophobicity of fluoroalkylsilane-and alkylsilane-grafted surfaces. Surf. Sci..

